# Short- and long-term transfer of urethral catheterization skills from simulation training to performance on patients

**DOI:** 10.1186/1472-6920-13-29

**Published:** 2013-02-25

**Authors:** Tobias Todsen, Mikael V Henriksen, Charles B Kromann, Lars Konge, Jesper Eldrup, Charlotte Ringsted

**Affiliations:** 1 Centre for Clinical Education, University of Copenhagen and The Capital Region of Denmark, Rigshospitalet Afsnit 5404, Teilumbygningen,Blegdamsvej 9, Copenhagen Ø DK-2100, Denmark; 2 Urology Department, Frederiksberg Hospital, University of Copenhagen and The Capital Region of Denmark, Copenhagen Ø, Denmark

## Abstract

**Background:**

Inexperienced interns are responsible for most iatrogenic complications after urethral catheterization (UC). Although training on simulators is common, little is known about the transfer of learned skills to real clinical practice. This study aimed to evaluate the short- and long-term effects of UC simulated skills training on performance on real patients and to examine whether watching a video of the procedure immediately before assessment enhanced clinical performance.

**Methods:**

This was an experimental study of the effect of a UC simulation-based skills course on medical students’ short-term (after one week) and long-term (after six weeks) performance. The additional effect of video instruction before performance testing on real patients was studied in a randomized trial. Sixty-four students participated in the study, which was preceded by a pilot study investigating the validity aspects of a UC assessment form.

**Results:**

The pilot study demonstrated sufficient inter-rater reliability, intra-class correlation coefficient 0.86, and a significant ability to discriminate between trainee performances when using the assessment form, p= 0.001. In the main study, more than 90% of students demonstrated an acceptable performance or better when tested on real patients. There was no significant difference in the total score between the one-week and the six-week groups when tested on real patients and no significant difference between the video and the control groups.

**Conclusions:**

Medical students demonstrated good transfer of UC skills learned in the skills lab to real clinical situations up to six weeks after training. Simulated UC training should be the standard for all medical school curricula to reduce avoidable complications. However, this study did not demonstrate that an instructional video, as a supplement to simulated skills training, improved clinical UC performance.

**Trial registration:**

Current Controlled Trials ISRCTN:ISRCTN90745002

## Background

Urethral catheterization (UC) is frequently performed on hospitalized patients, which makes it a core skill for any physician [1,2]. However, the procedure is associated with risks such as iatrogenic infection, false passage, paraphimosis, urethral strictures, and urethral trauma [3-6]. Previous research has shown that inexperienced interns are responsible for most iatrogenic complications [6]. The majority of complications can be prevented using the proper UC technique, and for patient safety, only well-trained personnel are recommended to perform UC [7]. However, interns believe they receive inadequate training [6] and there is currently no standard for adequate training [8].

Training procedural skills (such as UC) in simulation laboratories is common in many medical schools, yet skills training consumes considerable faculty teaching time and economic resources. One study demonstrated good results from UC skills training when assessed on a mannequin immediately after training [9]. However, little is known about the effect of simulation training in basic clinical skills on clinical performance [10]. A major concern with procedural skills training in a simulated setting is retention and transfer of the learned skills to real practice [11]. To transfer a skill successfully, students must learn to adjust their performance to the variety of conditions in real clinical situations [12,13]. Few studies have shown that simulation training improves participants’ performance on real patients [14,15], and no studies have investigated UC performance.

One barrier to retention and transfer of skills learned in simulation is the time it may take before a student has the opportunity to apply the skill in a clinical setting. As a result, long-term learning and patient safety may be threatened. However, to enhanced performance, students and junior doctors might watch an instructional video as a booster before performing clinical procedures [16]. An instructional video combined with simulation-based skills training has been shown to have good results on learning tested in simulated environments [17-21]. Despite this, there is no research on the effect of video instruction used in clinical settings immediately before procedural skills performance.

This study aimed to investigate the short-term and long-term effects of simulated UC skills training on performance on real patients and the effect of watching an instructional video of the UC procedure immediately before the first clinical performance.

## Methods

This experimental study included a main study of the short- and long-term effects of simulated UC skills training and the effect of additional video instruction. The study was preceded by a pilot study pilot study that investigated reliability and sensitivity of a UC assessment form for discriminating trainees' level of training.

### Pilot study

**Figure 1:**
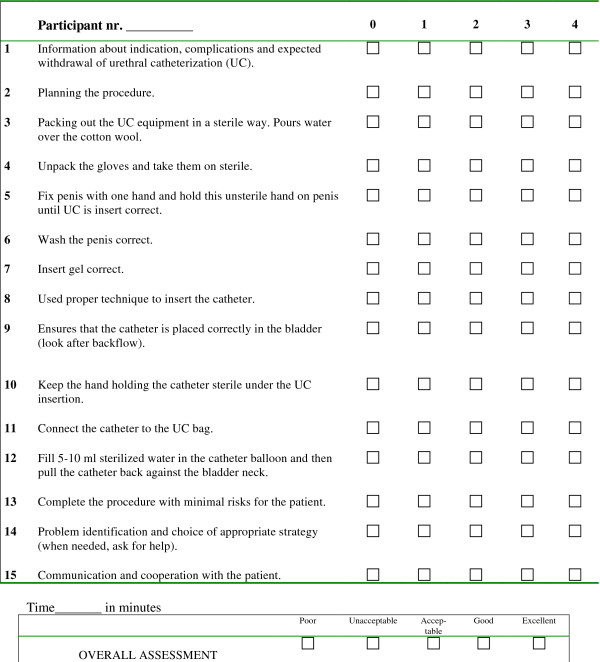
The assessment form in UC.

An experienced urologist, a UC skills teacher, and an educational researcher developed the assessment form (Figure 1), which was based on an objective structured clinical examination (OSCE) checklist previously used to assess UC skills [9] to which items concerning communication skills and patient safety were added (item number 1, 2, 13, 14 and 15). The assessment form had 15 items, each scored on a scale of 0–4, with 4 indicating the best performance. In addition, the form included an overall performance assessment rubric with five categories: poor, unacceptable, acceptable, good, and excellent.

Twenty-eight fourth-year medical students participated in the pilot study. All of the students had completed a simulated UC skills course two to four months earlier. Fifteen of the participants had never performed a UC on a patient (labelled: novices). Thirteen participants had performed UC on a patient at least once during their clinical clerkship (labelled: advanced beginners). All participants were videotaped while performing a UC on a male mannequin. An actor was sitting behind the mannequin’s lower body, acting as a patient with a need for UC. The students were told to act like the mannequin was a real patient and to communicate appropriately with the patient. Two experienced urology nurses assessed the students’ video-recorded performances independent of each other and blinded to the students’ previous UC experience. Before the pilot test the raters participated in a 90-min rater training session in which they assessed different test videos of UC performance and discussed their ratings until they reached consensus.

The inter-rater reliability of the assessments was explored by the intra-class correlation coefficient (ICC), single measures, consistency definition. The ability to discriminate between performance improvements was evaluated by comparing the mean score from the novices with the mean score from the advanced beginners using independent samples t-test.

### Main study

**Figure 2:**
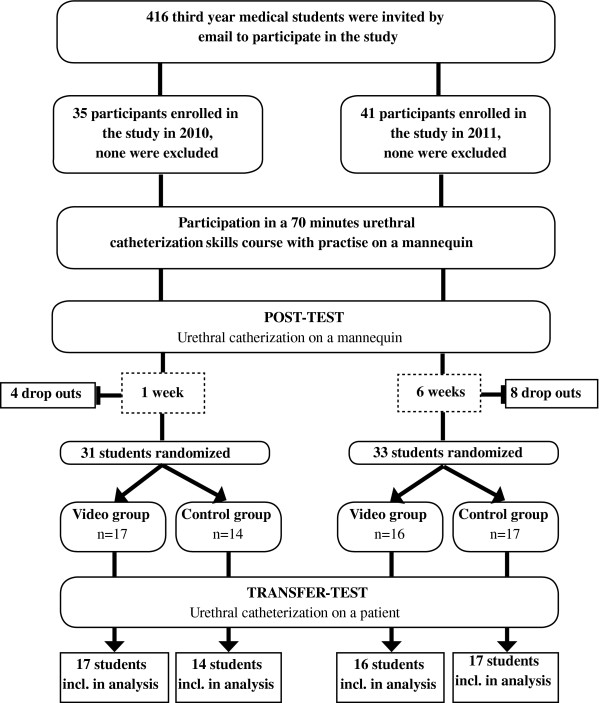
Flow diagram of the study.

Seventy-six medical students were enrolled in the main study between August 2010 and March 2011 (Figure 2). The participants were a volunteer sample of third-year medical students. All students were invited by email to participate in the study and were included on a first come, first served basis. Students with previous clinical experience in performing UC were excluded. All participants received a simulated skills training course covering the essential knowledge and skills needed to perform a UC. The course was highly standardized and conducted in a skills laboratory associated with the medical school. An experienced student teacher taught the students in groups of no more than six people. After a theoretical introduction, the skill was demonstrated and all students were allowed to practice once on a mannequin with feedback from the teacher. Immediately after the UC course, the students were post-tested using a scenario with a male mannequin like the one in the pilot study. Their performances were videotaped and assessed by a blinded rater, a physician who formerly taught UC in the skills lab.

Participants were scheduled to perform a UC on a patient from a urological department (transfer test) either one week or six weeks after completing the course. They were instructed not to practice UC skills during the delay. All patients wore urethral catheters permanently and had their urethral catheter changed at the hospital every two to three months. Patients with former UC difficulties or with mentally illness were excluded from the study. The Danish National Committee on Biomedical Research Ethics approved the study and verbal and written informed consent was obtained from the patients and medical students involved.

Twelve of the 76 students enrolled in the study dropped out after the skills course, most because their patients did not show up at the appointment time. Thirty-one participants were tested one week after the course (one-week group) during autumn 2010, and 33 were tested six weeks after the course (six-week group) during spring 2011. When the participants arrived at the hospital for the transfer test, they were located to a private room and randomized to a video group or a control group by a research fellow. All randomization sequences and tables were generated using http://www.random.org. The video group watched a five-minute video instruction of UC, while the control group did not get any preparation before the test. The video illustrated a physician performing UC on a real patient with a voice-over explaining the difficult steps. Afterwards, each participant performed a UC on a patient, and the procedure was assessed by direct observation by one of the two experienced urology nurses who participated in the pilot study. The nurses were blinded regarding randomization, and the participants’ performances were scored using the assessment form from the pilot study.

An independent samples t-test was used for comparison between groups and a paired sample t-test was used for comparison within groups. Differences were considered statistically significant when the p value was < 0.05. The effect size (ES) was estimated using Cohen’s d, with 0.2 representing a small ES, 0.5 a medium ES, and 0.8 a large ES [22]. The statistical analysis was performed using a statistical software package (PASW, version 18.0; SPSS Inc; Chicago IL).

## Results

### Pilot study

The inter-rater reliability was high, ICC = 0.86. The advanced beginners scored mean 42.1 points (SD=8.8), which were significantly higher than the novices’ 29.6 points (SD=8.0), p=0.001, Table 1.

**Table 1:** Demographics and total test score (average from two raters) of the medical students’ performance of UC in the pilot study

	**Novice group (n=15)**	**Advanced beginners group (n=13)**	**Difference between groups, independent samples t-test**
	**Mean (SD)**	**Mean (SD)**	
Age	24.9 (1.62)	24.1 (1.75)	P=0.192
Percentage of women	53.3	53.8	
Number of UC on real patients	0	5.69 (6.81)	P=0.003, ES=1.67
Total test score (0–60)	29.6 (8.04)	42.1 (8.77)	P= 0.001, ES=1.49

Standart deviation (*SD*), number of participants (*n*), P-value (*P*) and Effect Size (*ES*).

### Main study

All groups showed clinical significant effect from the UC skills course measured immediately after the course in the simulation setting (posttest) and after one or six weeks in clinical setting on real patients (transfer test). There was no significant difference in the total score between the posttest and the transfer test or between the one-week and the six-weeks groups (Table 2). Furthermore no significant differences were found between the control and video groups. Tested on real patients, 90.3% and 90.9% of the medical students in the control and video groups respectively demonstrated acceptable or better performance in the overall assessment scores.

**Table 2:** Medical students’ performance of UC skills immediately after a simulation course (posttest in simulation setting) and after one week or six weeks (transfer tests on real patients)

	**N**	**All**	**N**	**Video group**	**N**	**Control group**	**Difference between video- and control group, independent samples t-test**
		**Mean (SD)**		**Mean (SD)**		**Mean (SD)**	
**One week group**							
Posttest (Simulation setting)	31	46.0 (5.10)	17	45.4 (5.03)	14	46.6 (5.30)	P= 0.513
							ES= 0.233
Transfer test one week after training (real patients)	31	45.1 (10.3)	17	45.6 (8.89)	14	44.5 (12.1)	P= 0.763
							ES= 0.105
Difference between posttest and transfer test, Paired Samples T-Test	31	P= 0.898	17	P= 0.608	14	P= 0.676	
**Six weeks group**							
Posttest (simulation setting)	33	44.1 (7.30)	16	44.9 (6.12)	17	43.5 (8.40)	P= 0.572
							ES= 0.190
Transfer test six weeks after training (real patients)	33	46.7 (9.53)	16	48.6 (8.94)	17	44.9 (9.93)	P= 0.265
							ES= 0.391
Difference between posttest and transfer test, Paired Samples T-Test	33	P= 0.123	16	P= 0.168	17	= 0.440	
Difference between one week and six weeks, independent samples t-test		P= 0.520	33	P= 0.345	31	P= 0.924	
		ES= 0.162		ES= 0.337		ES= 0.0365	

Standart deviation (*SD*), number of participants (*n*), P-value (*P*) and Effect Size (*ES*).

Possible mean from 0 – 60.

## Discussion

The pilot study produced two sources of validity evidence including sufficient reliability [23] and distinguished between novices’ and advanced beginners’ performance with the use of the assessment form. The high ICC is attributed to the thorough rater training and the rather clear standards for performance. Hence, we suggest that the two sources of validity evidence supported our use of one rater for each assessment in the main study.

The results of the immediate posttest were similar to other studies [9], demonstrating that medical students benefited from the simulated skills training, with mean scores around 45 out of 60 possible. Medical students demonstrated poor performance of UC before participating in the simulation course [9], and participants in the study were likely to perform alike in a pretest. Consequently, we are confident in assuming that the students’ performance levels can be attributed to an effect of the UC skills course.

There was no difference in the mean score between the posttest and the transfer test, demonstrating that skills learned in simulation training were transferable to performance on real patients. More than 90% of the medical students demonstrated acceptable UC performance or better in the overall assessment scores on their first UC performance on a patient. We interpreted this as adequate performance after UC simulator training of novices. However, about 10% of the students still performed unacceptable UC and, therefore, we cannot recommend unsupervised clinical performance after completion of simulation training. For patient safety, initial UC simulation training should be incorporated into all medical school curricula. Barsuk et al. found a reduction in iatrogenic complications after implementation of simulated skills training in insertion of a central venous catheter [14]. This result may apply as well to other basic skills that interns are expected to perform independently. However, future studies on the effect of skills training on quality of care and patient safety are needed.

We found no significant difference between the group who watched the instructional video before performing UC on a real patient (video) and the control group. Previous studies reported ambiguous results. Some studies demonstrated a good effect of learning clinical skills from video instructions [16-21], while few others did not find any substantial effect [24,25]. Both groups in this study underwent simulation UC training and a posttest prior to the video randomization. The students in the control group received high ratings in the transfer test, and additional instructions might have been redundant. Due to ethical concerns, comparison to a group with no prior training was not feasible. Another reason that the video instruction showed no significant effect could be that the video group did not access the video freely, but were allowed to watch only once. Other studies showed that self-regulated control over instructions, including moving back and forth in the video, could enhance learning [26-28]. Furthermore, an effect size of 0.4 from the video intervention in the six-week group might indicate that a larger sample size could have resulted in a significant effect. Studies on how, why, and in which formats video instructions might contribute to learning skills are warranted and necessary.

## Conclusion

Medical students demonstrated good transfer of UC skills learned in the skills lab to real clinical situations up to six weeks after training. Simulated UC training should be the standard for all medical school curricula to reduce avoidable complications. However, this study did not demonstrate that an instructional video, as a supplement to simulated skills training, improved clinical UC performance.

## Abbreviation

UC: Urethral catheterization.

## Competing interests

The authors declare that they have no competing interests.

## Authors’ contributions

TT, LK, and CR participated in the design and the statistical analysis of the pilot study. TT, MJH, JE, CBK, and CR participated in the design of the main study. TT, MJH, and JE acquired the data and CBK made the interpretation. TT and CBK performed the statistical analysis of the main study. TT and LK have been involved in drafting the manuscript with CR as supervisor. All authors have critically read and revised the manuscript and all authors have approved the final manuscript.

## Pre-publication history

The pre-publication history for this paper can be accessed here:

http://www.biomedcentral.com/1472-6920/13/29/prepub
